# Nomograms based on lactate dehydrogenase to albumin ratio for predicting survival in colorectal cancer

**DOI:** 10.7150/ijms.71971

**Published:** 2022-05-29

**Authors:** Yugang Hu, Yanxiang Zhou, Yinghao Cao, Hao Wang, Yuanting Yang, Riyue Jiang, Qincheng Gong, Qing Zhou

**Affiliations:** 1 Department of Ultrasound Imaging, Renmin Hospital of Wuhan University, Wuhan, China; 430061; 2 Department of Gastrointestinal Surgery, Union Hospital, Tongji Medical College, Huazhong University of Science and Technology, Wuhan, China; 430022

**Keywords:** colorectal cancer, lactate dehydrogenase to albumin ratio, prognosis, inflammation, survival nomogram

## Abstract

**Purpose:** We aimed to determine if lactate dehydrogenase to albumin ratio (LAR) might play a prognostic role for patients with operable colorectal cancer (CRC).

**Patients and Methods:** 1334 operable CRC patients in Wuhan Union Hospital Between July 2013 and September 2017 were enrolled in this study and were randomly appointed them into training (n=954) and validation (n=380) sets. The relationship between LAR and overall survival (OS) and disease-free survival (DFS) were determined by restricted cubic splines (RCS) with Cox regression models. LAR was then divided into three categories based on the RCS and compared to the well-known TNM stage system. Finally, survival nomograms were developed by compounding the LAR and other clinical factors.

**Results:** Baseline LAR values and the all-cause mortality were U shaped, which slowly decreased until around 4.50 and then started to increase rapidly when the LAR ranged from 4.50-6.68 and then became flat thereafter (P for non-linearity <0.001). LAR was superior to TNM stage for OS as well as DFS and LAR plus TNM stage could add more net benefit than clinical model alone. Moreover, the survival nomograms based on LAR achieved great predictive ability for OS and DFS in operable CRC patients.

**Conclusions**: LAR could be served as a reliable prognostic factor for OS as well as DFS, with more accurate prognostic prediction than current TNM stage for patients with operable CRC.

## Introduction

Colorectal cancer (CRC) ranks as one of the most common solid tumors in patients all over the world 1-4. Despite great prognoses in our intelligence on its risk, development and especially in the field of surgical treatment and chemoradiotherapy, CRC remains a frustrating tumor without an optimistic long-term prognosis 5-7. Hence, determination of a great prognostic predictor with easily obtained and relatively good accuracy for a better risk stratification is essential for clinicians.

Lactate dehydrogenase (LDH) is a considerable indicator for liver in clinical practice and has been advocated to play a momentous role in immune condition in several cancers so as to CRC 8-13. Serum albumin is also a readily accostable element for nutrition and proposed to have prognostic importance for different tumor patients 14, 15. Therefore, the combination of them, LDH to serum albumin ratio (LAR), was recommended to be a good prognostic ratio for patients with cancers 16-18. However, limited study investigated the link between LAR and CRC patients 19. Therefore, in this study, we initially assessed the link between LAR and the outcomes of CRC patients. Then, a comparison between LAR and TNM stage system of predictive ability was also made. Finally, the survival nomograms based on LAR were built and verified in this study.

## Materials and methods

### Study population

We consecutively enrolled a CRC database with 3500 patients at the Wuhan Union Hospital from July 2013 and September 2017, as previously reported 20. In short, CRC patients without infective disease who did not receive anti-inflammatory agents prior to the surgical excision were studied. Patients with missing measurements of serum LDH or albumin before surgery treatments were also excluded. Finally, a total of 1334 CRC patients were studied and were randomly specified to the test (n=954) and verified sets (n=380).

This retrospective study was performed according to the Helsinki Declaration and all patients were asked to offers their informed consents. Moreover, the study was authorized by the Ethics Administration Office of our hospital.

### Data collection

Demographic data as well as other clinical information were automatically obtained from this platform. Initial laboratory results prior to the surgery treatment were also obtained. In addition, follow-up visits were implemented every 3 months in the first two years and twice a year in the following third to fifth years.

The primary outcome of this study was overall survival (OS) while the secondary outcome was disease-free survival (DFS).

The LAR was computed by initial serum LDH (U/L) /serum albumin (g/L).

### Statistical analyses

SPSS 23.0 and R 3.3.1 software were utilized for all analyses. Continuous variables were presented as means with standard deviations (SD) or interquartile ranges, and as frequencies along with percentages for binary variables. Multivariable Cox proportional hazards regression models were utilized to assess hazard ratios of overall mortality for LAR concentration. Firstly, we utilized restricted cubic spline (RCS) models fitted for Cox models with 5 knots at the 5th, 35th, 50th, 65th, and 95th percentiles of LAR. Through the RCS analysis, the cut-off values of the LAR based on OS were obtained, and then this continuous variable were translate to ternary variables in accordance with the cut-off values. Then, receiver operating characteristic (ROC) analyses, integrated discrimination index (IDI) and net reclassification improvement (NRI) were also exploited to assess the accuracy of LAR and TNM stage for clinical outcomes. Furthermore, LAR and other clinical features were also grouped to develop survival nomograms for OS and DFS. The discrimination and calibration were assessed by time-dependent ROC (td-ROC) curves and calibration curves, respectively. Finally, the decision curve analysis (DCA) was also utilized to assess the clinical benefits of the nomograms for OS and DFS. P < 0.05 at both sides represents that the difference is of statistical significance.

## Results

### Patient clinical features

A total of 1334 patients with CRC (803 men and 531 women) met the inclusion criterion were finally studied and patients were randomly assigned to the test (n=954) and verified sets (n=380). Furthermore, all variables in the test and verified sets were comparable in this study (Table 1).

### LAR and the risk of death and recurrence

**Figure 1:**
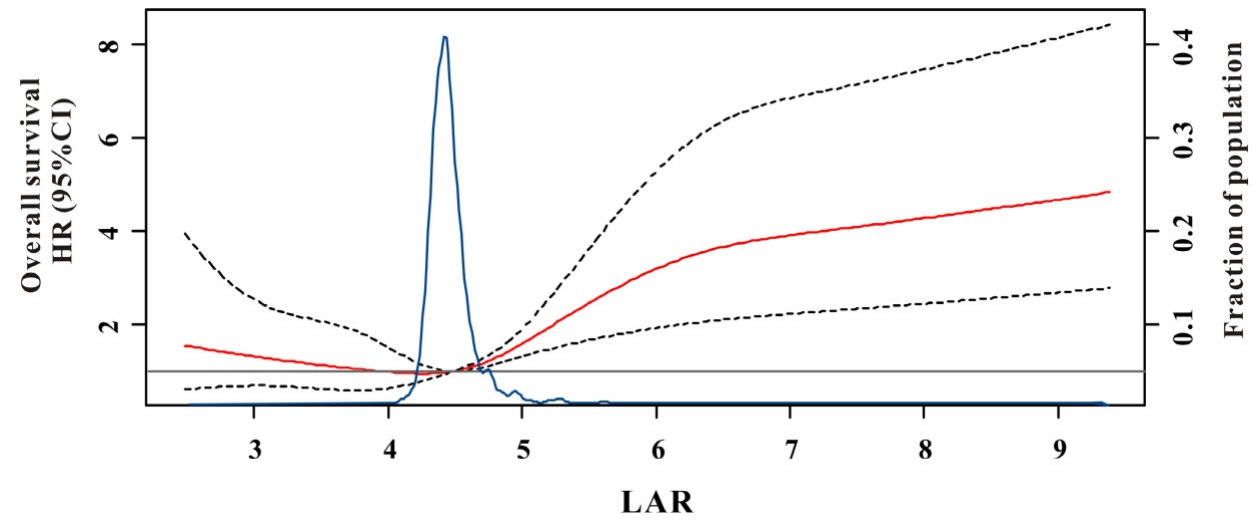
Multivariable adjusted hazard ratios (HR) for overall survival according to levels of lactate dehydrogenase to albumin ratio (LAR) on a continuous scale. Solid red lines are multivariable adjusted HR, with dashed black lines showing 95% confidence intervals derived from restricted cubic spline regressions with three knots. Reference lines for no association are indicated by the solid gray lines at a hazard ratio of 1.0. Dashed blue curves show the fraction of the population with different levels of LAR.

**Figure 2:**
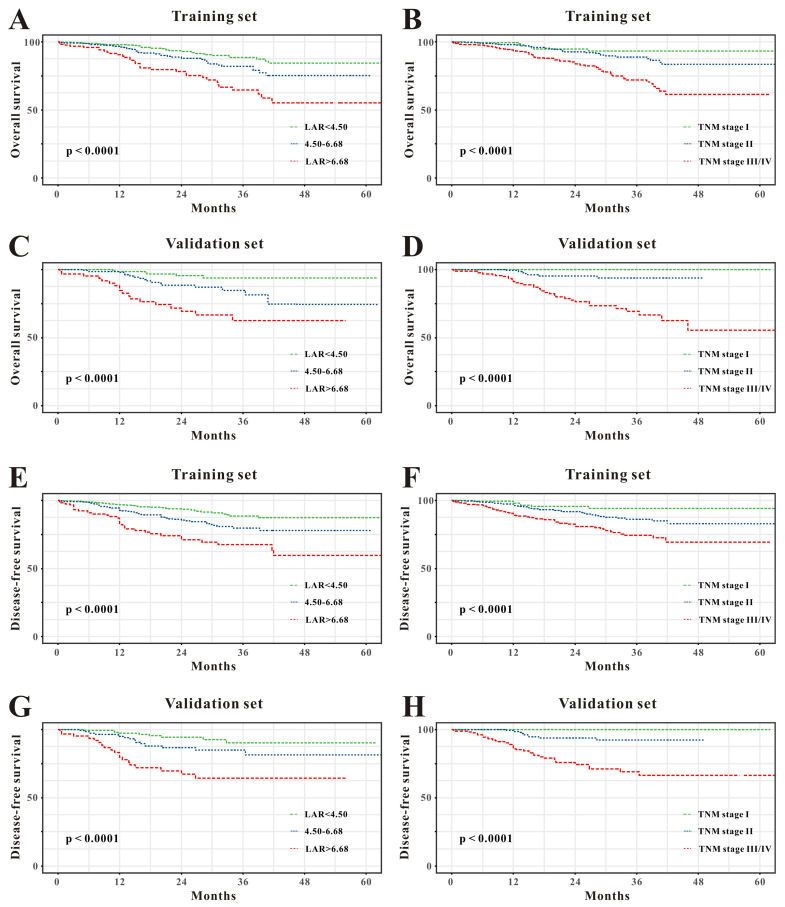
Kaplan-Meier survival curves for overall survival (OS) (**A-D**) and disease-free survival (DFS) (**E-H**) of the test cohort and the verified cohort in different models. LAR is divided into high-risk group, intermediate, and low-risk group in the training cohort (**A** and** C**) and in the validation cohort (**E** and** G**); TNM staging system is divided into stage I, stage II and stage III/IV in the training cohort (**B** and** D**) and in the validation cohort (**F** and** H**).

During the median of 21.9 months (ranges 0.2-79.0 months) for OS and 21.2 months (ranges 0.2-75.0 months) for DFS follow-up, we found 114 (11.9%) deaths and 119 (12.5%) recurrences in the test set. RCS showed a U-curved link between LAR and OS after adjusted confounding indexes (Figure 1). In Figure 2, the risk of all-cause mortality was slowly decreasing until around 4.50 of predicted OS and then started to increase rapidly when the LAR was ranged from 4.50-6.68 and then became flat thereafter (P for non-linearity <0.001). Moreover, a similar result had also been conducted for the relationship between LAR and DFS in the test cohort Supplemental Figure 1. Therefore, according to the results of RCS, we classified CRC patients into three categories based on the values of LAR: low-risk (<4.5), intermediate (4.5-6.68) and high-risk group (>6.68).

### The comparison of LAR and TNM staging system

Compared with patients in the low-risk group, patients with CRC in the high-risk or intermediate groups revealed worse OS and DFS in the training cohort (Figure 2A and E) so as to in the verified set (Figure 2C and G). Similarly, TNM system showed significantly different survival probability between stages in the training set (Figure 2B and F) and in the verified cohort (Figure 2D and H). Moreover, the accuracy of LAR for OS was 0.708 and 0.714, respectively. Similarly, LAR also obtained relatively good capability for DFS (AUC=0.706, 0.712, respectively, Table 2). Meanwhile, the AUCs of the TNM stage system for OS were 0.642 and 0.653, respectively, while for DFS, AUCs were 0.644 and 0.676, respectively. Furthermore, as described in Table 2, the addition of LAR significantly improved the risk reclassification (as measured using NRI and IDI) of OS as well as DFS compared to the TNM stage system. Therefore, LAR demonstrated better predicting performance of the OS and DFS than TNM stage system in patients with CRC.

### Construction and validation of survival nomograms

**Figure 3:**
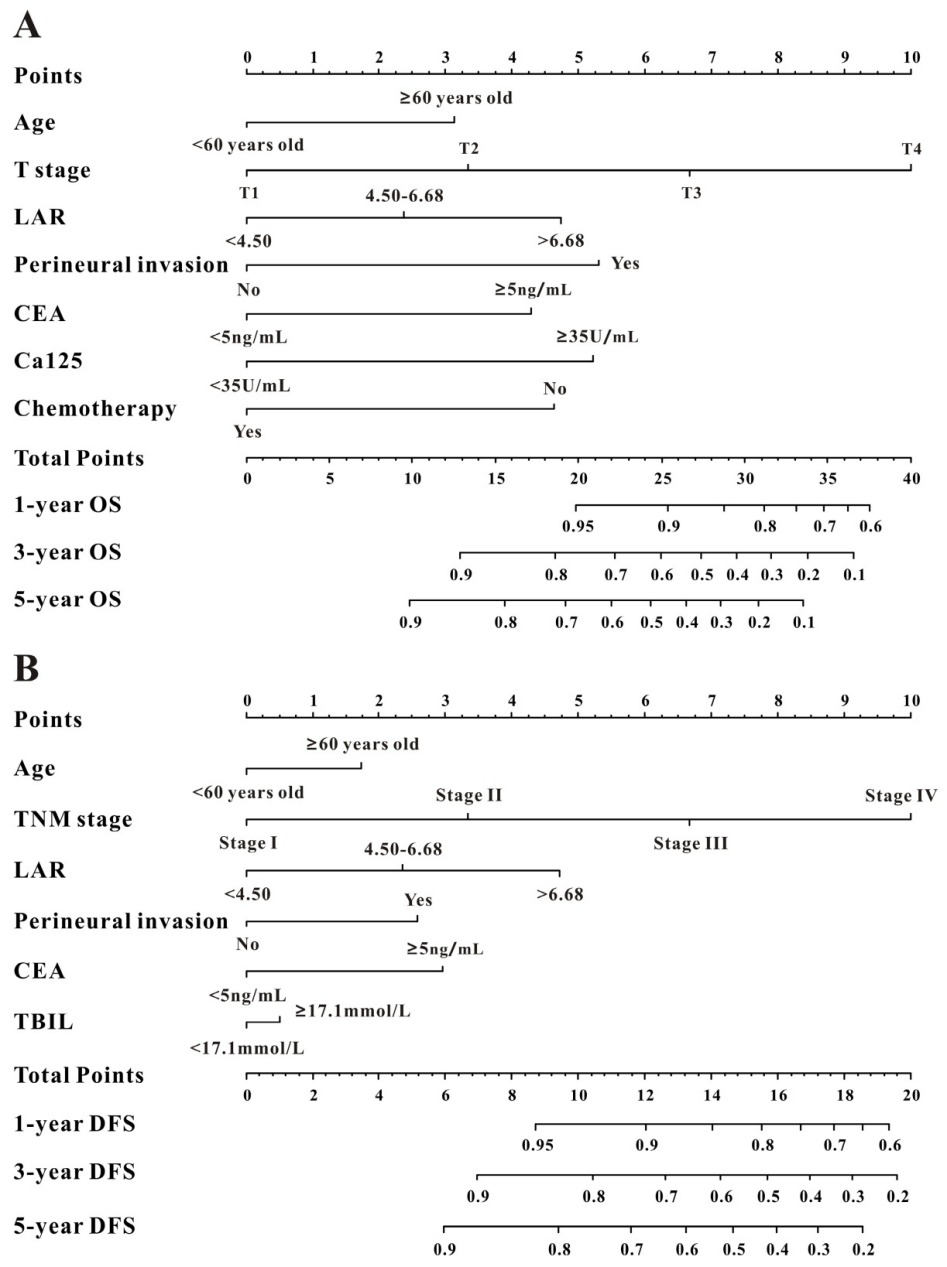
Evaluation of overall survival (**A**) and disease-free survival (**B**) associated nomograms for operable patients with CRC.

**Figure 4:**
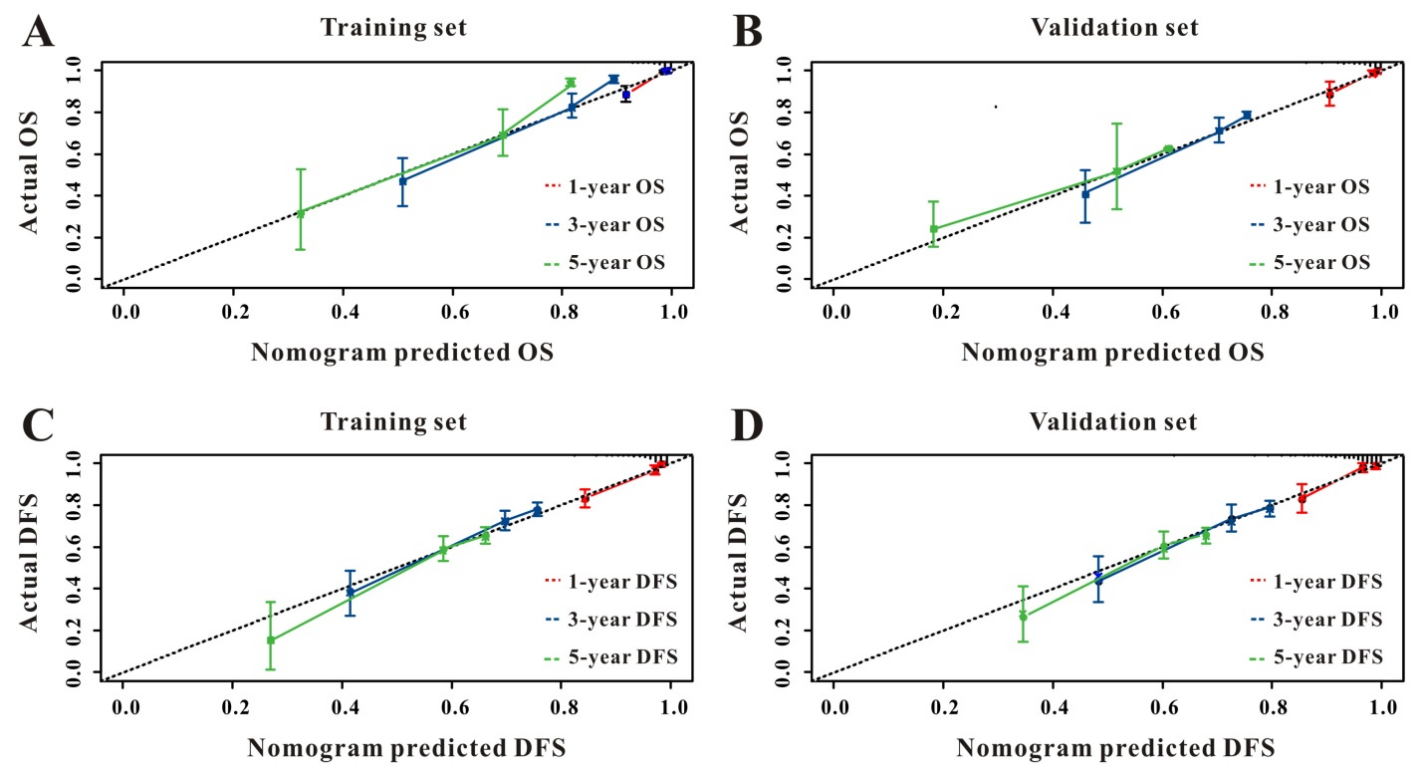
The calibration curves for predicting OS in CRC patients at 1-, 3-, and 5-year in the test set (**A**) and at 1-, 3-, and 5-year in the verified set (**B**). The calibration curves for predicting DFS in CRC patients at 1-, 3-, and 5-year in the test set (**C**) and at 1-, 3-, and 5-year in the verified set (**D**).

**Figure 5:**
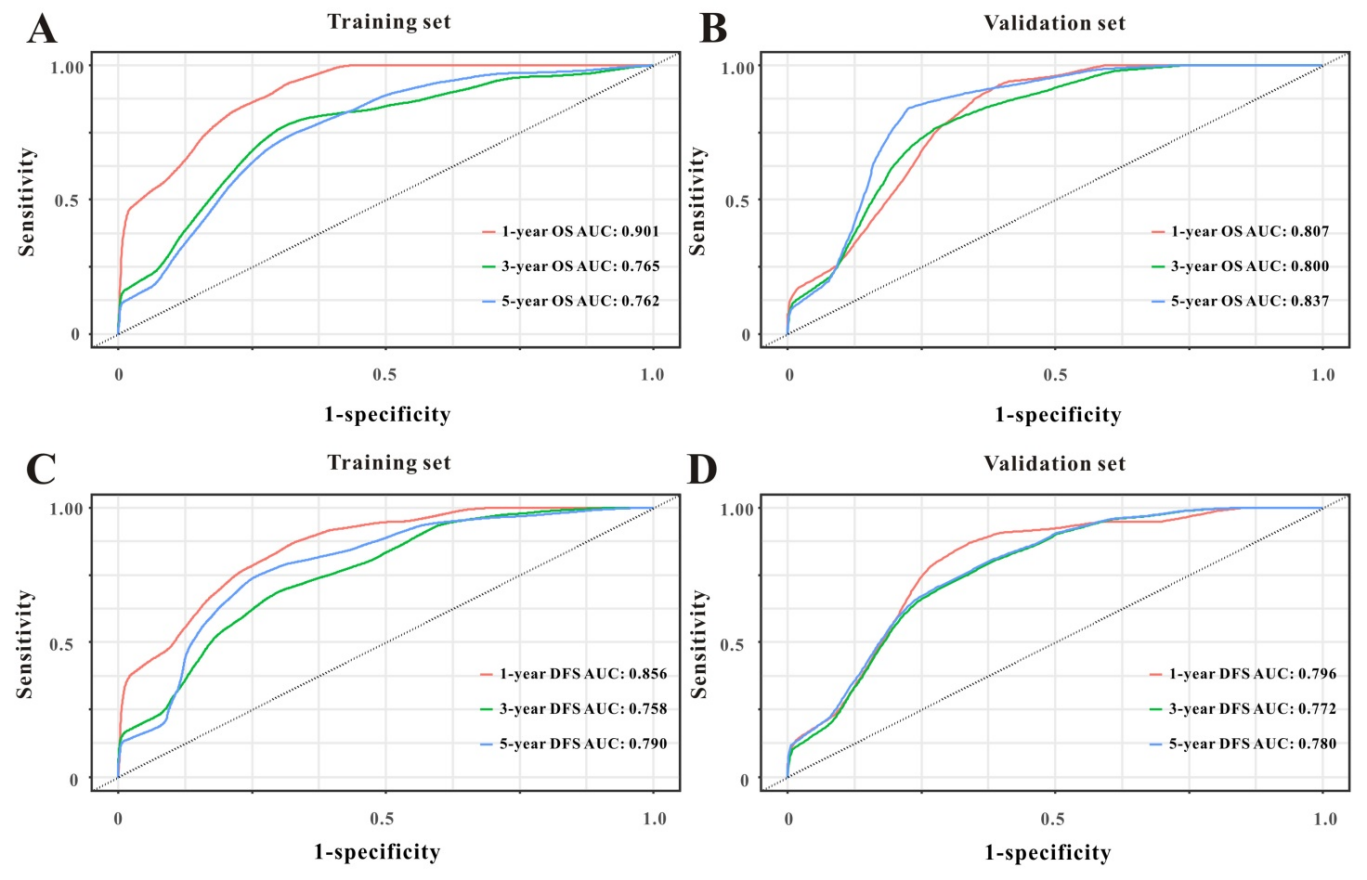
Time-dependent ROC curves from the nomograms for the prediction of OS and DFS in the test (**A, C**) and verified (**B, D**) sets, respectively.

**Figure 6:**
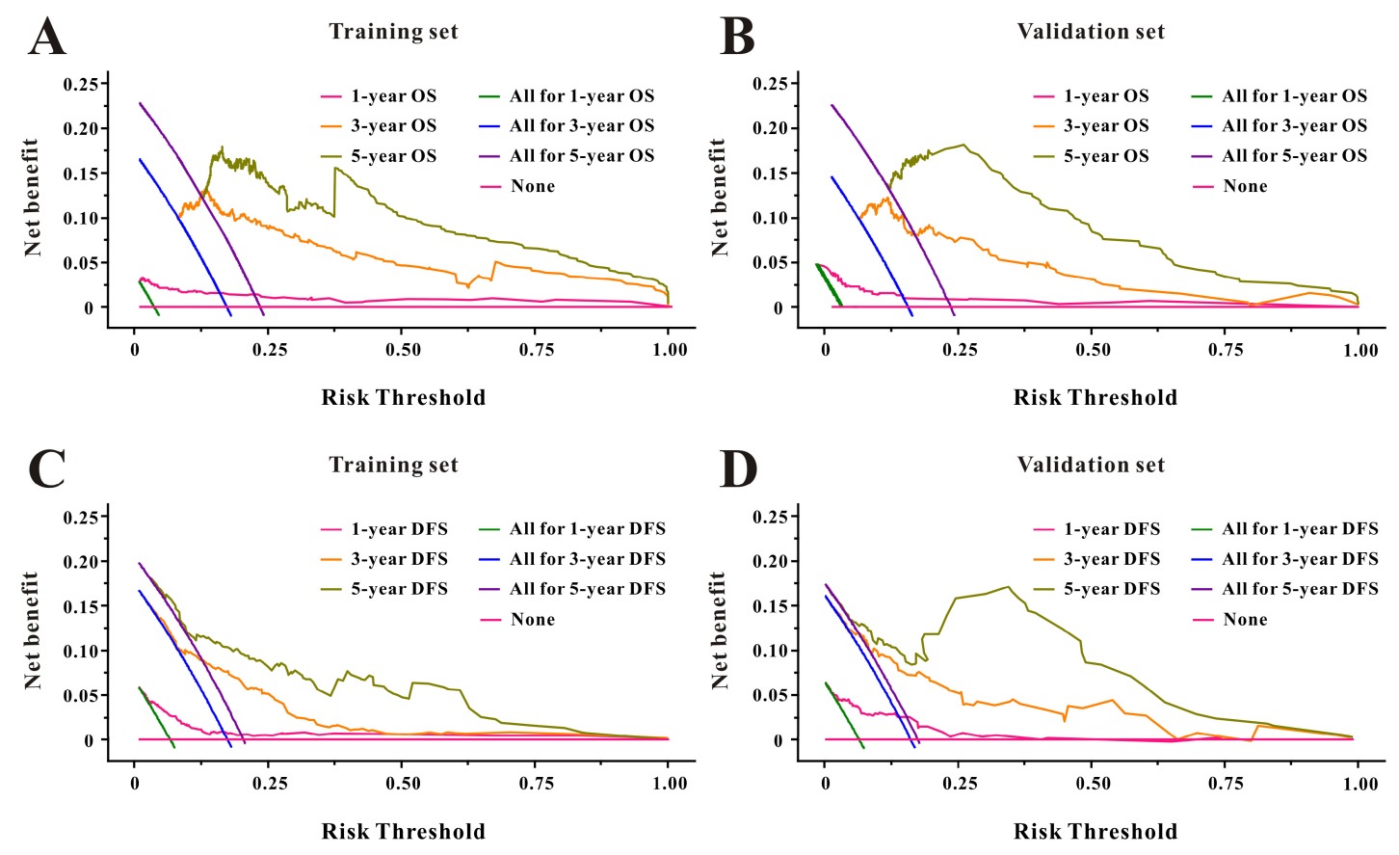
Decision curve analysis of LAR for overall survival and disease-free survival in CRC patients to detect its clinical usefulness in the training set (**A, C**), and in the validation set (**B, D**), respectively.

Based on the multivariate Cox results for OS Supplemental Table 1, seven variables were finally included in the OS nomogram: age, LAR, T stage, perineural invasion, CEA, CA125 and chemotherapy (Figure 3A). The calibration curves (Figure 4A-B) revealed that the prognostic nomogram possessed responsible reproducibility. What's more, td-ROC analyses were also utilized to assess the fatidic value for OS and DFS. In the evaluation of the 1-year, 3-year and 5-year survival rates, the predictive power measured by AUCs were 0.901, 0.765 and 0.762 in the test set (Figure 5A), and 0.807, 0.800 and 0.837 in the verified set (Figure 5B), respectively. According to the DCA, when the threshold probability for a patient was within the range of 0-100%, the nomogram added more net benefit than the “treat all” or “treat none” strategies both in the test cohort and in the verified cohort for 1-, 3-, 5-year OS (Figure 6A-B), which indicated that the prognostic nomogram could be clinical usefulness for OS with CRC patients.

As for DFS Supplemental Table 2, six informative variables (age, LAR, TNM stage, perineural invasion, CEA, total bilirubin) were eventually incorporated into the establishment of the DFS nomogram (Figure 3B). First, the calibration curves (Figure 4C-D) indicating that the prognostic nomogram for DFS possessed reliable repeatability. Then, in the assessment of the 1-year, 3-year and 5-year survival rates, the predictive power of the survival nomogram as measured by AUCs were 0.856, 0.758 and 0.790 in the test set (Figure 5C), and 0.796, 0.772 and 0.780 in the verified set (Figure 5D), respectively. Finally, the nomogram added more net benefit than the “treat all” or “treat none” strategies both in the test cohort and in the verified cohort for DFS (Figure 6C-D) according to DCA curves, which indicated that the survival nomogram could be clinical usefulness for DFS for CRC patients.

## Discussion

In this retrospective study of 1334 individuals, we demonstrated a U-shaped relationship between LAR and all-cause mortality and recurrence in CRC patients who received surgical excision treatment. Based on the results of RCS, LAR values were converted into ternary variables and compared with the current TNM stage system, LAR was superior to TNM stage for predicting OS and DFS and LAR plus TNM stage could added more net benefit for OS and DFS than clinical model alone. Moreover, the survival nomograms incorporating LAR and other clinical features reached the much higher predictive value in predicting OS and DFS in CRC patients than any other single factor. Therefore, our study concluded that the LAR could serve as a reliable prognostic marker for OS and DFS in patients with operable CRC.

Despite recent advancement in the diagnosis and treatment of them, CRC remains to be the second most common reason for cancer death. Even with radical resection as well as neoadjuvant chemoradiotherapy, the rates of 5-year OS for CRC patients remain pessimistic, with approximately 70% for stage II and 60% for stage III 21. Hence, it's important for clinicians to determine CRC patients at high risk of mortality with easily accessible and cost-effective biomarker.

It's well known that inflammation plays a significant role in the initial, development and prognosis of cancer patients and thus inflammation-based methods could be applied to assess the prognosis of cancer patients 22, 23. Though hematoxylin- eosin (H&E)-stained slices based on the morphology characteristics of inflammatory cells is reliable to evaluate the cancer-associated inflammation, it is inconvenient and invasive 24. Hence, most clinicians had concentrated on the link between inflammatory indexes and prognosis of CRC patients 25-27. Among them, LAR might be one of the most relevant biomarkers for CRC patients.

The link between LAR and clinical outcomes of cancer patients has been conducted in several studies 16-18. Peng et al. revealed that the LAR played an important role for poor OS (HR=1.60, 95%CI 1.23-2.10, P=0.001) and progression-free survival (HR=1.42, 95%CI 1.10-1.85, P=0.008) in a retrospective study of 1661 patients with nasopharyngeal carcinoma when the cutoff value was reached at 4.04 28. Feng et al. divided LAR into two categories based on X-tile and concluded that LAR is a helpful potential prognostic biomarker for surgical esophageal squamous cell carcinoma patients with the optimal cut-off value was 5.5 29. Moreover, a recent retrospective study with 295 CRC patients undergoing curative resection revealed that higher LAR (≥ 52.7) was importantly combined with worse OS and DFS 19. Similarly, in this study, we firstly found a U shaped relationship between baseline LAR and prognosis of CRC patients, in addition, we divided the LAR into three categories and compared with the currently TNM system, LAR was superior to TNM stage for predicting OS and DFS and LAR plus TNM stage could added more net benefit for OS and DFS than clinical model alone.

LAR is the ratio of LDH concentration and serum albumin value. LDH had also been proposed to reflect tumor growth, invasive potential and immune suppression 30, 31 and had been demonstrated to have interpersonal link with prognosis in several cancers especially gastrointestinal tract cancers 32-34. In cancer patients, low level of serum albumin means the status of malnutrition, which may lead to decreased synthesis and lateral leakage and thus serum albumin had also been proposed to have interpersonal link with the prognosis of several malignant tumors 35, 36. LDH and serum albumin are clinical availability and the combination of them, LAR, is not only a marker of inflammation but also reflect nutritional condition, however, limited data is accessible for the combination of LDH and serum albumin for the prognosis of patients with CRC. In the current study, we firstly found an non-linear relationship between LAR levels and the prognosis of CRC patients undergoing curative resection. Moreover, when we combined LAR with other significant variables to create survival nomograms, we found that the survival nomograms obtained great predictive ability in the prediction of OS or DFS, implying that LAR may act as a new prognostic factor for patients with CRC.

There are some limitations in this study. Firstly, this was a retrospective study carried out in a single center and lacks external verification. Secondly, we only collected the initial levels of LAR prior to surgical treatment and dynamic monitoring of LAR during hospital stay may be more precise in this way. Finally, we did not conduct the gene profiling related to inflammatory pathways considering the lack of related medical records. Hence, large scale prospective cohort studies are needed in more patients with different cancer species to determine the broader independent predictive effects of LAR and to external verify our survival nomograms.

## Conclusions

Our study firstly found the non-linear relationship between LAR and the prognosis of CRC patients and demonstrated that LAR seems to be a promising marker of survival outcomes in operable CRC patients. LAR outperforms the well-known TNM stage system and LAR plus TNM stage could added more net benefit for OS and DFS than clinical model alone. Moreover, nomograms based on LAR for predicting OS and DFS in CRC may serve as a clinically personalized tool to provide reliable prognostic information for the greatest survival benefits for CRC patients through layered management.

## Supplementary Material

Supplementary figures and tables.

## Figures and Tables

**Table 1:** Clinicopathological characteristics of all patients

Characteristics	Training set (n=954)	Validation set (n=380)	P value
Age (years) (mean (SD))	58.5±11.9	58.2±12.4	0.685
Sex, male, n (%)	557 (58.4)	246 (64.7)	0.038
BMI, kg/m^2^ (mean (SD))	22.8±2.9	22.7±2.9	0.758
Smoking, n (%)	220 (23.1)	97 (25.5)	0.377
Family history of cancer, n (%)	83 (8.7)	34 (8.9)	0.971
Primary site, n (%)			0.992
Left colon	238 (24.9)	96 (25.3)	
Right colon	225 (23.6)	89 (23.4)	
Rectum	491 (51.5)	195 (51.3)	
Histological grade, n (%)			0.276
Well differentiated	135 (14.2)	66 (17.4)	
Moderately differentiated	683 (71.6)	257 (67.6)	
Poorly differentiated	136 (14.3)	57 (15.0)	
Tumor size, n (%)			0.267
<2cm	53 (5.6)	14 (3.7)	
2-5cm	672 (70.4)	265 (69.7)	
≥5cm	229 (24.0)	101 (26.6)	
Perineural invasion, n (%)			0.05
Yes	187 (19.6)	84 (22.1)	
No	767 (80.4)	296 (77.9)	
T stage, n (%)			0.478
T1	69 (7.2)	21 (5.5)	
T2	158 (16.6)	59 (15.5)	
T3	546 (57.2)	217 (57.1)	
T4	181 (19.0)	83 (21.9)	
N stage, n (%)			0.903
N1	550 (57.7)	222 (58.4)	
N2	254 (26.6)	102 (26.8)	
N3	150 (15.7)	56 (14.7)	
TNM stage, n (%)			0.708
Stage I	168 (17.6)	60 (15.8)	
Stage II	370 (38.8)	159 (41.8)	
Stage III	405 (42.5)	158 (41.6)	
Stage IV	11 (1.2)	3 (0.8)	
Adjuvant chemotherapy, n (%)			0.698
Yes	490 (51.4)	190 (50.0)	
No	464 (48.6)	190 (50.0)	
Post radiotherapy, n (%)			0.059
Yes	39 (4.1)	24 (6.3)	
No	915 (95.9)	356 (93.8)	
Laboratory results, median (IQR)			
WBC, ×10^9^/L	6.0 (5.0, 7.0)	6.0 (5.0, 7.0)	0.137
HGB, g/dL	121.5 (104.0, 136.0)	120.0 (102.8, 134.0)	0.309
PLT, ×10^9^/L	221.5 (178.0, 277.0)	220.0 (172.0, 277.0)	0.655
Albumin, g/L	40.0 (36.0, 43.0)	40.0 (36.8, 43.0)	0.907
Bilirubin, mmol/L	11.0 (8.0, 15.0)	11.0 (8.0, 14.0)	0.794
ALP, U/L	74.0 (62.0, 89.0)	73.0 (60.0, 85.0)	0.121
LDH, U/L	184.0 (157.0, 195.0)	182.0 (157.3, 191.0)	0.894
LAR, U/g	4.5 (3.9, 5.2)	4.5 (3.9, 5.2)	0.830
Creatinine, umol/L	69.0 (59.0, 80.8)	70.0 (61.0, 82.0)	0.207
Urea nitrogen, mmol/L	5.0 (4.0, 6.0)	5.0 (4.0, 6.0)	0.374
CEA, ng/mL	4.0 (2.0, 8.0)	4.0 (2.0, 9.0)	0.834
CA125, U/mL	12.0 (8.0, 18.0)	12.0 (8.0, 18.0)	0.983
CA199, U/mL	8.5 (4.0, 22.8)	8.0 (3.0, 20.0)	0.262
Overall survival months	21.9 (14.0, 33.4)	21.9 (13.7, 32.0)	0.433
Disease-free survival months	21.2 (13.4, 32.9)	21.3 (13.4, 31.7)	0.482
Death, n (%)	114 (11.9)	42 (11.1)	0.715
Recurrence, n (%)	119 (12.5)	46 (12.1)	0.926

BMI, body mass index, IQR, interquartile range, WBC, white blood count, HGB, hemoglobin, PLT, platelet, ALP, alkaline phosphatase, LDH, lactate dehydrogenase, LAR, lactate dehydrogenase to albumin ratio, CEA, carcino-embryonic antigen, CA125, carbohydrate antigen 125.

**Table 2:** NRI and IDI analyses for risk reclassification of overall survival and disease-free survival

Outcome			AUC			IDI		NRI^a^	
	Sensibility (%)	Specificity (%)	Biomarker	Biomarker+ clinical model	clinical model^b^	Value (95%CI)	P Value	Value (95%CI)	P Value
In training set									
For OS									
LAR	60.4	79.6	0.708	0.807	0.797	0.013 (0.002-0.026)	0.039	0.100 (0.030-0.170)	0.005
TNM stage	65.8	59.4	0.642	0.799		0.003 (-0.002-0.008)	0.232	0.056 (0.002-0.111)	0.042
LAR+TNM	60.3	80.8	0.728	0.810		0.017 (0.003-0.031)	0.020	0.112 (0.038-0.185)	0.003
For DFS									
LAR	58.9	75.5	0.706	0.776	0.784	0.017 (0.003-0.032)	0.020	0.109 (0.031-0.188)	0.006
TNM stage	63.0	59.2	0.644	0.762		0.009 (0.001-0.017)	0.027	0.095 (0.021-0.169)	0.012
LAR+TNM	59.6	82.3	0.734	0.795		0.027 (0.011-0.044)	<0.001	0.163 (0.080-0.247)	<0.001
In validation set									
For OS									
LAR	57.1	80.6	0.714	0.826	0.812	0.011 (0.004-0.017)	0.031	0.103 (0.040-0.305)	0.011
TNM stage	63.3	62.7	0.653	0.815		-0.001 (-0.017-0.014)	0.862	-0.006 (-0.018-0.006)	0.317
LAR+TNM	64.3	84.7	0.734	0.833		0.016 (0.005-0.037)	0.024	0.164 (0.032-0.296)	0.015
For DFS									
LAR	56.4	79.7	0.712	0.813	0.794	0.019 (0.006-0.047	0.009	0.118 (0.052-0.265)	0.004
TNM stage	66.4	67.9	0.676	0.803		0.008 (-0.014-0.030)	0.464	-0.011 (-0.109-0.087)	0.822
LAR+TNM	67.0	86.4	0.746	0.821		0.033 (0.007-0.059)	0.005	0.173 (0.048-0.338)	0.009

AUC, area under the receiver-operating characteristic curve, IDI, integrated discrimination improvement, NRI, Net reclassification index, OS, overall survival, LAR, lactate dehydrogenase to albumin ratio, DFS, disease-free survival. ^a^The NRI is calculated through two-way category by using the event rate of overall survival and disease-free survival.^b^The clinical model for predicting overall survival and disease-free survival are composed of age, gender, BMI, smoking, family history of cancer, primary site, tumor size, grade, T stage, N stage, M stage, perineural invasion, chemotherapy, radiotherapy, and laboratory results except for LAR and albumin.
