# Supplementary material for: Nomograms based on lactate dehydrogenase to albumin ratio for predicting survival in colorectal cancer
Source: Int J Med Sci. 2022 May 29;19(6):1003–12. doi: 10.7150/ijms.71971 (PMC9254365; doi:10.7150/ijms.71971)
Supplement: Supplementary file 1 — Supplementary figures and tables. [file ijmsv19p1003s1.pdf]

Supplemental Table 1. Univariate and multivariate COX analyses of factors associated with OS in training set

	Univariate analysis		Multivariate analysis	
	HR (95%CI)	P	HR (95%CI)	P
Age	1.04 (1.03-1.06)	<0.001	1.02 (1.01-1.04)	0.022
Sex, male	0.74 (0.50-1.09)	0.128		
BMI	0.94 (0.89-1.01)	0.072		
Smoking	0.74 (0.46-1.20)	0.223		
Family history of cancer	0.47 (0.17-1.27)	0.136		
Primary site				
Left colon	1.40 (0.90-2.18)	0.133		
Right colon	1.41 (0.90-2.19)	0.140		
Rectum	Ref.	-		
Histological grade				
Well differentiated	Ref.	-		
Moderately differentiated	0.74 (0.43-1.28)	0.283		
Poorly differentiated	1.59 (0.87-2.90)	0.135		
Tumor size				
<2cm	Ref.	-		
2-5cm	2.06 (0.65-6.53)	0.219		
≥5cm	2.76 (0.85-9.00)	0.092		
Perineural invasion				
Yes	2.61 (1.73-3.93)	<0.001	1.76 (1.13-2.76)	0.013
No	Ref.	-	Ref.	-
T stage				
T1	Ref.	-	Ref.	-
T2	2.36 (0.53-10.46)	0.258	1.95 (0.43-8.75)	0.384
T3	3.64 (0.89-14.89)	0.072	2.68 (0.55-13.00)	0.222
T4	7.10 (1.71-29.46)	0.007	4.15 (1.84-16.54)	0.038
N stage				
N1	Ref.	-	Ref.	-
N2	2.27 (1.48-3.48)	<0.001	0.77 (0.18-3.43)	0.737
N3	3.33 (2.09-5.32)	<0.001	1.18 (0.27-5.22)	0.831
TNM stage				
Stage I	Ref.	-	Ref.	-
Stage II	1.63 (0.75-3.54)	0.219	0.85 (0.29-2.51)	0.767
Stage III	4.10 (1.97-8.52)	<0.001	2.28 (0.38-13.85)	0.370
Stage IV	9.62 (2.89-32.07)	<0.001	2.81 (0.62-12.79)	0.083
Adjuvant chemotherapy				
Yes	0.45 (0.30-0.67)	<0.001	0.56 (0.36-0.86)	0.009
No	Ref.	-	Ref.	-
Post radiotherapy				
Yes	0.48 (0.12-1.94)	0.302		
No	Ref.	-		

Laboratory results				
WBC	1.08 (1.03-1.14)	0.003	1.01 (0.95-1.09)	0.683
HGB	0.99 (0.98-1.00)	0.082		
PLT	1.00 (0.99-1.02)	0.090		
Bilirubin	1.02 (1.01-1.04)	0.006	1.01 (0.98-1.03)	0.567
ALP	1.01 (1.00-1.02)	0.002	1.00 (1.00-1.02)	0.587
LAR				
<4.50	Ref.	-	Ref.	-
4.50-6.68	1.88 (1.24-2.86)	0.003	1.61 (1.04-2.50)	0.034
>6.68	4.48 (2.62-7.69)	<0.001	3.19 (1.97-5.17)	<0.001
Creatinine	1.00 (0.99-1.01)	0.610		
Urea nitrogen	1.10 (1.00-1.20)	0.049	0.99 (089-1.10)	0.882
Lg (CEA)	2.37 (1.88-2.97)	<0.001	1.40 (1.02-1.93)	0.035
Lg (CA125)	3.43 (2.28-5.16)	<0.001	1.78 (1.06-2.99)	0.030
Lg (CA199)	1.92 (1.52-2.44)	<0.001	1.24 (0.97-1.59)	0.091

OS, overall survival, BMI, body mass index, WBC, white blood count, HGB, hemoglobin, PLT, platelet, ALP, alkaline phosphatase, LAR, lactate dehydrogenase to albumin ratio, CEA, carcino-embryonic antigen, CA125, carbohydrate antigen 125.

Supplemental Table 2. Univariate and multivariate COX analyses of factors associated with DFS in training set

	Univariate analysis		Multivariate analysis	
	HR (95%CI)	P	HR (95%CI)	P
Age	1.04 (1.02-1.06)	<0.001	1.02 (1.01-1.04)	0.027
Sex, male	0.78 (0.54-1.14)	0.205		
BMI	0.98 (0.92-1.04)	0.500		
Smoking	0.86 (0.55-1.35)	0.516		
Family history of cancer	0.30 (0.10-1.16)	0.142		
Primary site				
Left colon	1.54 (0.99-2.36)	0.051		
Right colon	1.42 (0.91-2.22)	0.119		
Rectum	Ref.	-		
Histological grade				
Well differentiated	Ref.	-		
Moderately differentiated	0.71 (0.42-1.19)	0.195		
Poorly differentiated	1.39 (0.77-2.51)	0.271		
Tumor size				
<2cm	Ref.	-		
2-5cm	2.31 (0.73-7.32)	0.153		
≥5cm	2.55 (0.78-8.35)	0.121		
Perineural invasion				
Yes	2.54 (1.70-3.78)	<0.001	1.64 (1.05-2.56)	0.029
No	Ref.	-	Ref.	-
T stage				
T1	Ref.	-	Ref.	-
T2	2.70 (0.61-11.86)	0.190	1.77 (0.39-8.03)	0.458
T3	3.63 (0.89-14.88)	0.073	2.40 (0.50-11.45)	0.273
T4	8.59 (2.08-35.46)	0.003	3.41 (0.71-16.36)	0.126
N stage				
N1	Ref.	-	Ref.	-
N2	1.68 (1.09-2.58)	0.018	0.94 (0.27-3.31)	0.924
N3	2.92 (1.87-4.55)	<0.001	1.44 (0.40-5.12)	0.578
TNM stage				
Stage I	Ref.	-	Ref.	-
Stage II	2.25 (1.00-5.06)	0.050	1.12 (0.37-3.36)	0.847
Stage III	4.36 (2.00-9.52)	<0.001	1.51 (0.28-8.14)	0.630
Stage IV	39.09(14.44-105.84)	<0.001	8.11 (1.96-33.62)	0.004
Adjuvant chemotherapy				
Yes	0.57 (0.39-0.83)	0.003	0.74 (0.49-1.11)	0.143
No	Ref.	-	Ref.	-
Post radiotherapy				
Yes	0.67 (0.21-2.12)	0.500		
No	Ref.	-		

Laboratory results				
WBC	1.07 (1.04-1.14)	0.003	1.01 (0.96-1.08)	0.703
HGB	0.99 (0.99-1.00)	0.114		
PLT	1.01 (0.99-1.01)	0.074		
Bilirubin	1.03 (1.01-1.05)	0.003	1.02 (1.01-1.04)	0.007
ALP	1.01 (1.00-1.01)	0.015	1.00 (1.00-1.01)	0.890
LAR				
<4.50	Ref.	-	Ref.	-
4.50-5.88	2.27 (1.50-3.44)	<0.001	1.80 (1.15-2.81)	0.010
>5.88	4.79 (2.76-8.34)	<0.001	2.14 (1.24-3.71)	0.006
Creatinine	0.99 (0.98-1.00)	0.307		
Urea nitrogen	1.04 (1.02-1.06)	0.389		
Lg (CEA)	2.41 (1.93-3.01)	<0.001	1.49 (1.10-2.03)	0.011
Lg (CA125)	2.80 (1.84-4.27)	<0.001	1.13 (0.67-1.90)	0.643
Lg (CA199)	2.04 (1.62-2.56)	<0.001	1.26 (0.98-1.62)	0.074

DFS, disease-free survival, BMI, body mass index, WBC, white blood count, HGB, hemoglobin, PLT, platelet, ALP, alkaline phosphatase, LAR, lactate dehydrogenase to albumin ratio, CEA, carcino-embryonic antigen, CA125, carbohydrate antigen 125.

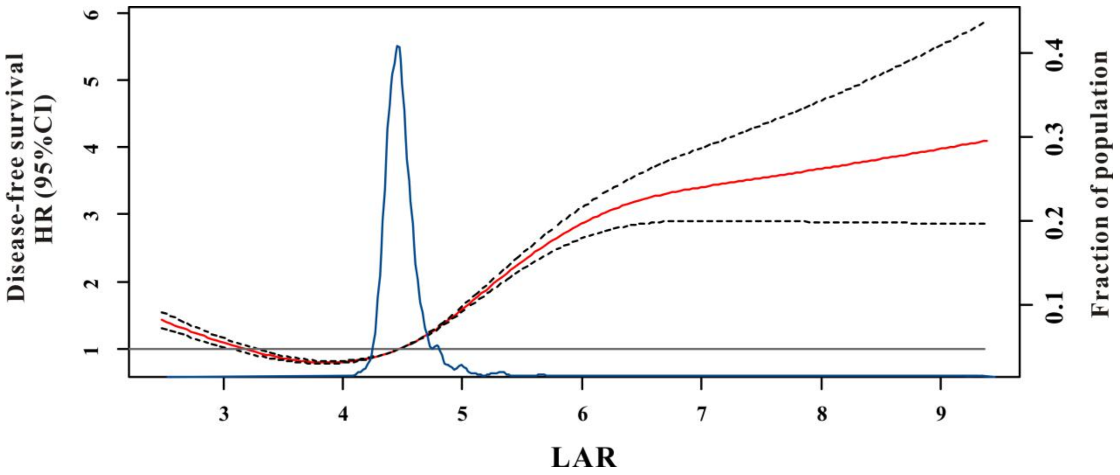

Supplemental Figure 1 Multivariable adjusted hazard ratios (HR) for disease-free survival according to levels of lactate dehydrogenase to albumin ratio (LAR) on a continuous scale. Solid red lines are multivariable adjusted HR, with dashed black lines showing 95% confidence intervals derived from restricted cubic spline regressions with three knots. Reference lines for no association are indicated by the solid gray lines at a hazard ratio of 1.0. Dashed blue curves show the fraction of the population with different levels of LAR.
